# Association between HIV and incident pulmonary hypertension in US Veterans: a retrospective cohort study

**DOI:** 10.1016/s2666-7568(21)00116-1

**Published:** 2021-06-16

**Authors:** Meredith S Duncan, Charles W Alcorn, Matthew S Freiberg, Kaku So-Armah, Olga V Patterson, Scott L DuVall, Kristina A Crothers, Vincent Lo Re, Adeel A Butt, Joseph K Lim, Joon Woo Kim, Hilary A Tindle, Amy C Justice, Evan L Brittain

**Affiliations:** Department of Biostatistics, University of Kentucky, Lexington, KY, USA; Division of Cardiovascular Medicine, Vanderbilt University Medical Center, Nashville, TN, USA; Department of Biostatistics, Graduate School of Public Health, University of Pittsburgh, Pittsburgh, PA, USA; Division of Cardiovascular Medicine, Department of Medicine, Vanderbilt University Medical Center, Nashville, TN, USA; Geriatric Research Education and Clinical Centers (GRECC), Veterans Affairs Tennessee Valley Healthcare System, Nashville, TN, USA; Section of General Internal Medicine, Boston University School of Medicine, Boston, MA, USA; VA Informatics and Computing Infrastructure, Department of Veterans Affairs Salt Lake City Health Care System, Salt Lake City, UT, USA; Department of Internal Medicine, Division of Epidemiology, University of Utah School of Medicine, Salt Lake City, UT, USA; VA Informatics and Computing Infrastructure, Department of Veterans Affairs Salt Lake City Health Care System, Salt Lake City, UT, USA; Department of Internal Medicine, Division of Epidemiology, University of Utah School of Medicine, Salt Lake City, UT, USA; Department of Medicine, Division of Pulmonary, Critical Care, and Sleep Medicine, University of Washington, Seattle, WA, USA; Veterans Affairs Puget Sound Healthcare System, Seattle, WA, USA; Division of Infectious Diseases, Department of Medicine, Perelman School of Medicine, University of Pennsylvania, Philadelphia, PA, USA; Veterans Affairs Pittsburgh Healthcare System, Pittsburgh, PA, USA; Weill Cornell Medical College, New York, NY, USA; Weill Cornell Medicine-Qatar, Doha, Qatar; Yale Liver Center and Section of Digestive Diseases, Yale University, New Haven, CT, USA; Department of Medicine, James J Peters VA Medical Center, Icahn School of Medicine at Mt Sinai, New York, NY, USA; Department of Medicine, Vanderbilt University Medical Center, Nashville, TN, USA; Geriatric Research Education and Clinical Centers (GRECC), Veterans Affairs Tennessee Valley Healthcare System, Nashville, TN, USA; Yale School of Medicine, Yale University, New Haven, CT, USA; Department of Medicine, Veterans Affairs Connecticut Healthcare System, West Haven, CT, USA; Division of Cardiovascular Medicine, Department of Medicine, Vanderbilt University Medical Center, Nashville, TN, USA

## Abstract

**Background:**

Pulmonary hypertension incidence based on echocardiographic estimates of pulmonary artery systolic pressure in people living with HIV remains unstudied. We aimed to determine whether people living with HIV have higher incidence and risk of pulmonary hypertension than uninfected individuals.

**Methods:**

In this retrospective cohort study, we evaluated data from participants in the Veterans Aging Cohort Study (VACS) referred for echocardiography with baseline pulmonary artery systolic pressure measures of 35 mm Hg or less. Incident pulmonary hypertension was defined as pulmonary artery systolic pressure higher than 35 mm Hg on subsequent echocardiogram. We used Poisson regression to estimate incidence rates (IRs) of pulmonary hypertension by HIV status. We then estimated hazard ratios (HRs) by HIV status using Cox proportional hazards regression. We further categorised veterans with HIV by CD4 count or HIV viral load to assess the association between pulmonary hypertension risk and HIV severity. Models included age, sex, race or ethnicity, prevalent heart failure, chronic obstructive pulmonary disease, hypertension, smoking status, diabetes, body-mass index, estimated glomerular filtration rate, hepatitis C virus infection, liver cirrhosis, and drug use as covariates.

**Findings:**

Of 21 314 VACS participants with at least one measured PASP on or after April 1, 2003, 13 028 VACS participants were included in the analytic sample (4174 [32%] with HIV and 8854 [68%] without HIV). Median age was 58 years and 12 657 (97%) were male. Median follow-up time was 3·1 years (IQR 0·9–6·8) spanning from April 1, 2003, to Sept 30, 2017. Unadjusted IRs per 1000 person-years were higher in veterans with HIV (IR 28·6 [95% CI 26·1–31·3]) than in veterans without HIV (IR 23·4 [21·9–24·9]; p=0·0004). The risk of incident pulmonary hypertension was higher among veterans with HIV than among veterans without HIV (unadjusted HR 1·25 [95% CI 1·12–1·40], p<0·0001). After multivariable adjustment, this association was slightly attenuated but remained significant (HR 1·18 [1·05–1·34], p=0·0062). Veterans with HIV who had a CD4 count lower than 200 cells per μL or of 200–499 cells per μL had a higher risk of pulmonary hypertension than did veterans without HIV (HR 1·94 [1·49–2·54], p<0·0001, for those with <200 cell μL and HR 1·29 [1·08–1·53], p=0·0048, for those with 200–499 cells per μL). Similarly, veterans with HIV who had HIV viral loads of 500 copies per mL or more had a higher risk of pulmonary hypertension than did veterans without HIV (HR 1·88 [1·46–2·42], p<0·0001).

**Interpretation:**

HIV is associated with pulmonary hypertension incidence, adjusting for risk factors. Low CD4 cell count and high HIV viral load contribute to increased pulmonary hypertension risk among veterans with HIV. Thus, as with other cardiopulmonary diseases, suppression of HIV should be prioritised to lessen the burden of pulmonary hypertension in people living with HIV.

**Funding:**

National Heart, Lung, and Blood Institute (National Institutes of Health, USA); National Institute on Alcohol Abuse and Alcoholism (National Institutes of Health, USA).

## Introduction

Small studies have suggested that people living with HIV have increased incidence of pulmonary arterial hypertension compared with those without HIV.^1,2^ Previous studies with echocardiography data examining the association between HIV and pulmonary hypertension in large cohorts have been cross-sectional and have therefore been unable to establish temporality between HIV infection and development of pulmonary hypertension. Thus, longitudinal studies are needed to establish whether HIV is associated with incident pulmonary hypertension and whether higher levels of detectable HIV viraemia increase the risk of pulmonary hypertension.

To answer these questions, we used data from the Veterans Aging Cohort Study (VACS), a large contemporary cohort of veterans with HIV and of demographically similar veterans without HIV infection with serial measurements of pulmonary artery systolic pressure (PASP) and robust data on comorbidities.^3^ This study builds on our previous work assessing prevalent pulmonary hypertension in VACS by using serial measurements of PASP measured by echocardiography extracted from medical records using natural language processing tools.^4^ In our analyses, pulmonary hypertension is defined using data from echocardiogram reports rather than from International Classification of Disease (ICD) codes,^5^ which have poor sensitivity and specificity. Although we do not define pulmonary hypertension using gold-standard haemodynamic data, the use of echocardiographic data is highly relevant to this population because most patients with WHO Group II or III pulmonary hypertension never undergo right heart catheterisation. As a result, pulmonary hypertension is necessarily defined using echocardiography in these patients. In this study, our aim was to determine whether HIV had a direct effect on incident pulmonary hypertension and PASP progression outside of its indirect effect through heart failure and chronic obstructive pulmonary disease (COPD).^6^ We also sought to determine whether the risk of pulmonary hypertension increased with worsening HIV infection (using time-varying metrics of HIV viraemia), and whether this risk differed by demographics such as sex and race or ethnicity.

## Methods

### Study design and participants

In this retrospective cohort study, we used data from VACS participants who had had a clinical echocardiogram and in whom an estimated PASP was reported. VACS is a longitudinal electronic health record cohort of veterans with HIV, of whom each veteran is matched to two veterans without HIV (1:2) on the basis of age, race or ethnicity, sex, year of enrolment, and clinical site.^3^ Cohort data sources include Veteran Affairs (VA) electronic health records, VA fee-for-service documentation, and Centers for Medicare and Medicaid Services.

Baseline was defined as the date of first echocardiogram with an estimated PASP on or after April 1, 2003. Included VACS participants had to have a first PASP of 35 mm Hg or less, non-zero follow-up time, and no change in HIV serostatus during the study. There were no age criteria for inclusion. The institutional review boards at Tennessee Valley Healthcare System, Vanderbilt University Medical Center (Nashville, TN, USA); Yale University (New Haven, CT, USA), and West Haven VA Medical Center (West Haven, CT, USA) approved this study.

### Data collection

We extracted echocardiographic data from all 2D echocardiograms performed in the VA system between April 1, 2003, and Sept 30, 2017 (inclusive) on VACS participants.^4^ We obtained estimated PASP measurements from Doppler echocardiography via a previously validated natural language processing tool applied to the VA electronic medical record.^7^ We used the calculated PASP estimate when reported. When tricuspid regurgitant velocity and estimated right atrial pressure were reported we calculated PASP using the simplified Bernoulli equation. When only the tricuspid regurgitant velocity was reported we assumed a right atrial pressure of 5·5 mm Hg, the median value in our cohort, which is a conservative assumption and is consistent with previous population-based studies.^8-10^ To ensure the quality of our data, we manually reviewed 100 charts to validate PASP measurements and excluded PASP estimates considered to be outside a physiological range (<8 mm Hg or >159 mm Hg).

### Exposure, outcomes, and covariates

Ascertainment of HIV infection status in the VACS cohort has been previously described.^3^ Briefly, HIV status was determined on the basis of two criteria: (1) at least one inpatient or two outpatient ICD, 9th revision (ICD-9) codes for AIDS (042), asymptomatic HIV (V08), or related diagnostic-related group codes (488–490) in the VA medical record, and (2) the participant was included in the VA Immunology Case Registry.^3^

The primary outcome was incident pulmonary hypertension. We defined incident pulmonary hypertension as a post-baseline PASP higher than 35 mm Hg, which corresponds to a mean pulmonary artery pressure of 21 mm Hg. We selected this definition to be consistent with the pulmonary hypertension definition adopted at the 6th World Symposium on Pulmonary Hypertension and because values lower than 40 mm Hg are consistently associated with adverse outcomes.^11-15^ Thus, our definition aligns with the contemporary invasive definition of pulmonary hypertension and is supported by previous literature. Follow-up time for individuals who developed pulmonary hypertension was defined as the date from baseline echocardiogram to the date of PASP estimate higher than 35 mm Hg. For individuals who did not develop pulmonary hypertension, including those without a subsequent PASP after baseline, follow-up time was defined as the date of first echocardiogram until the date of death or Sept 30, 2017.

In secondary analyses, we examined two other time-to-event outcomes. The first used a cutoff of 40 mm Hg to define pulmonary hypertension; the second examined PASP progression, defined as an increase in PASP of more than 10 mm Hg from baseline PASP. There is no established threshold for a clinically meaningful progression in PASP because previous studies have been cross-sectional. Importantly, a difference in 10 mm Hg in cross-sectional studies is associated with a substantial increase in risk of mortality.^8,16^

Age, sex, race or ethnicity, prevalent heart failure, COPD, hypertension, smoking, diabetes, body-mass index (BMI), estimated glomerular filtration rate (eGFR), liver cirrhosis (represented by fibrosis-4 score [FIB-4]), hepatitis C virus (HCV) infection, and recreational drug use were included as covariates. Variable definitions and methods of ascertainment are detailed in the appendix. To handle missing covariate data, we generated 20 complete datasets using multiple imputation by chained equation techniques. Specifically, we implemented predictive mean matching to produce biologically plausible values.^17^ Results from each imputed dataset were combined according to Rubin’s rules.^18^

### Statistical analysis

We generated descriptive statistics by HIV status as mean, standard deviation, median, and IQR for continuous variables and as counts and proportions for categorical variables. Differences in baseline characteristics were tested using Wilcoxon rank sum tests for continuous variables and χ^2^ tests for categorical variables.

Using all PASP estimates obtained during follow-up, we plotted the mean PASP in each year by HIV status to assess the longitudinal changes of PASP during follow-up. We used Poisson regression to estimate pulmonary hypertension incidence rates (IRs) per 1000 person-years and corresponding 95% CIs by HIV status; veterans with HIV were further categorised by their baseline CD4 count (≥500, 200–499, or <200 cells per μL) or HIV1-RNA (<500 or ≥500 copies per mL). After confirming that the proportional hazards assumption was met by inspecting log-log survival plots, we performed Cox proportional hazards regression to estimate the hazard ratios (HRs) of incident pulmonary hypertension in veterans with HIV compared with veterans without HIV. We further categorised veterans with HIV by CD4 count or HIV viral load to assess the association between risk of pulmonary hypertension and HIV severity. Cox proportional hazards regression models were unadjusted and then adjusted for age, sex, race or ethnicity, prevalent heart failure, COPD, hypertension, smoking, diabetes, BMI, eGFR, liver cirrhosis, HCV infection, and recreational drug use. Variable definitions and methods of ascertainment are detailed in the online data supplement. For adjusted models with HIV viral load or CD4 count stratification, we repeated the analysis viral load or CD4 count data as time-varying covariates. This means that each participant contributed person-time to the viral load or CD4 count category reflecting their status every time these HIV biomarkers were measured. For individuals who experienced pulmonary hypertension, the event contributed to the group in which the participant held membership on the date of the echocardiogram indicating pulmonary hypertension.

We also sought to examine other predictors of incident pulmonary hypertension included in our multivariable models. First, we included two-way interaction terms between HIV status and each covariate in Cox proportional hazards regression models to determine whether the association between the covariate and incident pulmonary hypertension differed by HIV status. We then fit a multivariable-adjusted Cox proportional hazards regression model including HIV, all covariates, significant interaction terms between HIV and covariates identified in the former step, and CD4 count and HIV viral load as conditionally relevant predictors.^19^

Baseline PASP was added to our primary models to assess robustness of our results. In additional sensitivity analyses, incident pulmonary hypertension models were limited to individuals without a history of heart failure or COPD at baseline, adjusting for interim heart failure or COPD as time-varying covariates to assess the association between HIV and incident pulmonary hypertension in the absence of these drivers of pulmonary hypertension. Since different thresholds are used to define pulmonary hypertension in clinical practice, additional sensitivity analyses altered the definition of incident pulmonary hypertension as a subsequent PASP higher than 40 mm Hg. Secondary analyses repeated all primary survival analyses outlined in this section with PASP progression of more than 10 mm Hg as the outcome.

All analyses were performed in SAS 9.4 (Cary, NC, USA) and a two-sided p value of less than 0·05 was considered significant.

### Role of the funding source

The funder of the study had no role in study design, data collection, data analysis, data interpretation, or writing of the report.

## Results

From 21 314 VACS participants with at least one measured PASP on or after April 1, 2003, we excluded 8096 individuals with prevalent pulmonary hypertension (first PASP >35 mm Hg regardless of their PASP on follow-up echocardiograms), 30 participants with zero follow-up time, and 160 people with documented changes in HIV serostatus during the study. The final analytic sample included 13 028 VACS participants (4174 [32%] with HIV and 8854 [68%] without HIV) with an estimated PASP lower than 35 mm Hg at baseline (median baseline PASP was 28 mm Hg [IQR 23–31]; median PASP measurements per person was 2 [1–3]). 12 657 (97%) of 13 028 individuals were male and the median age was 58 years (IQR 52–64; table 1). 6309 [48%] of 13 028 participants were African American, and 5235 (40%) were white. Prevalent COPD was common (3957 [30%]) whereas heart failure was uncommon at the time of the baseline PASP estimate (272 [2%]). While most characteristics were similar between veterans with HIV and those without HIV, it differed for HCV infection and diabetes. Of veterans with complete data for HCV infection, 1373 (34%) of 4014 veterans with HIV had HCV infection compared with 1504 (18%) of 8439 veterans without HIV; and 1163 (28%) of 4174 veterans with HIV had diabetes compared with 3494 (39%) of 8854 veterans without HIV. 873 (21%) of 4174 veterans with HIV were antiretroviral therapy (ART) naive at baseline while 2632 (63%) were currently on ART (median time on ART 8·2 years [IQR 3·3–13·1]). Median CD4 count among veterans with HIV was 443 cells per μL (IQR 261–661). 1958 (55%) of 3573 veterans with HIV with complete data for baseline viral load had undetectable viral load (≤50 copies per mL); accordingly, median HIV viral load was 50 copies per mL (IQR 40–400).

**Figure 1:**
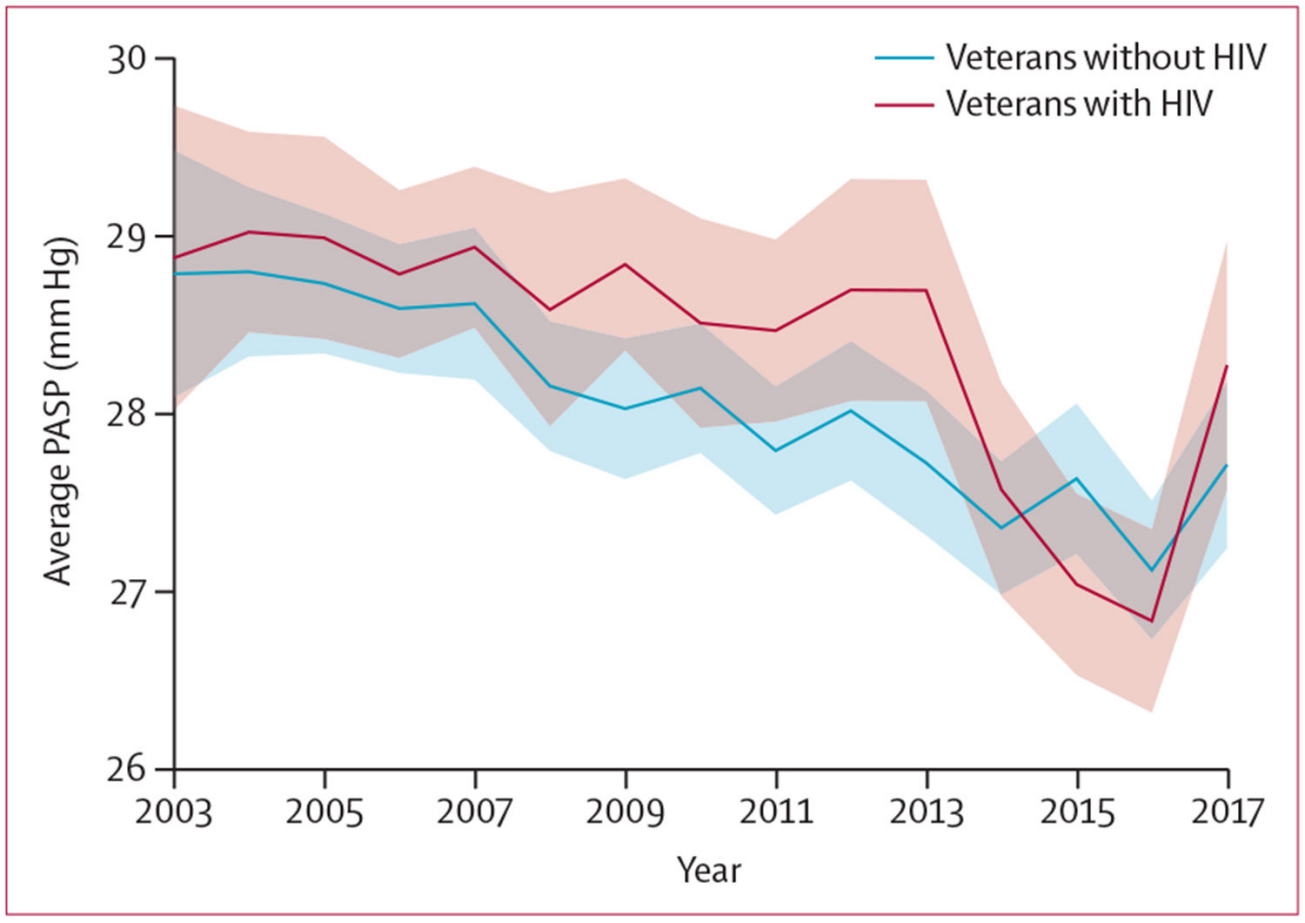
Changes in PASP over time by HIV status Mean PASP by HIV status. Shaded intervals are corresponding 95% CIs. PASP=pulmonary artery systolic pressure.

During a median follow-up of 3·3 years (IQR 1·0–7·1) in veterans without HIV and 2·8 years (IQR 0·8–6·4) in veterans with HIV, spanning from April 1, 2003, to Sept 30, 2017, there were 1372 incident pulmonary hypertension events (468 in veterans with HIV and 904 in veterans without HIV). From the plot of mean PASP per year by HIV status, we noted that although mean PASP in 2003 was similar by HIV status, veterans with HIV tended to have higher mean PASP over time (figure 1). However, the 95% CIs surrounding mean PASP overlapped for the two groups throughout follow-up, and general trends in increasing and decreasing PASP over time were similar between groups (figure 1).

**Figure 2:**
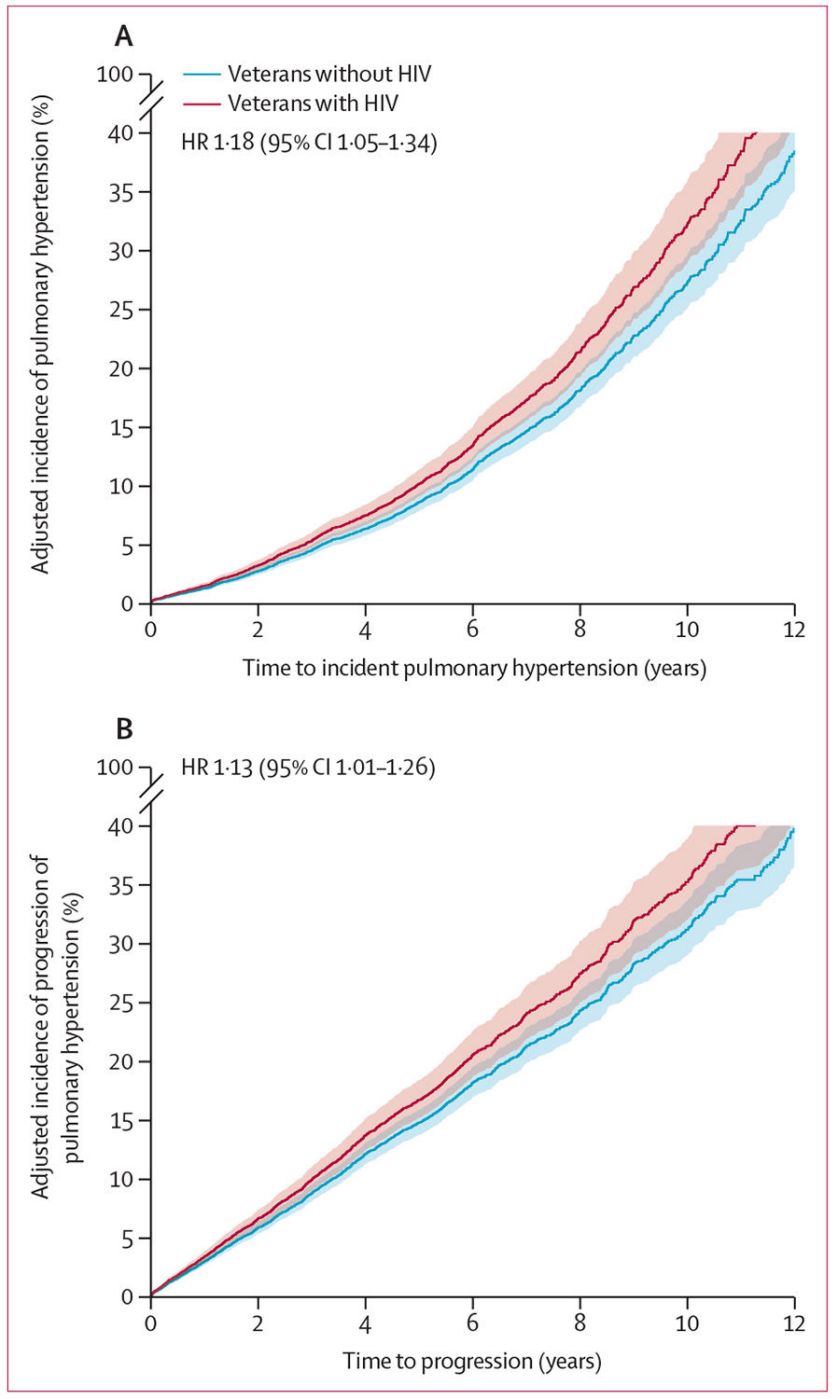
Adjusted incidence of pulmonary hypertension (A) and PASP progression (B) Multivariable adjusted models include age, sex, race or ethnicity, prevalent heart failure, chronic obstructive pulmonary disease, hypertension, diabetes, smoking status, body-mass index, eGFR, liver cirrhosis, hepatitis C virus infection, and recreational drug use as covariates. PASP=pulmonary artery systolic pressure. eGRF=estimated glomerular filtration rate. HR=hazard ratio.

Unadjusted IRs per 1000 person-years were higher in veterans with HIV (IR 28·6 [95% CI 26·1–31·3]) than in veterans without HIV (IR 23·4 [21·9–24·9]; p=0·0004). The risk of incident pulmonary hypertension was higher among veterans with HIV than among veterans without HIV (unadjusted HR 1·25 [95% CI 1·12–1·40], p<0·0001). After multivariable adjustment, this association was slightly attenuated but remained significant (HR 1·18 [1·05–1·34], p=0·0062; figure 2A).

In multivariable adjusted models, veterans with HIV who had baseline HIV viral loads of 500 copies per mL or higher had significantly higher risk of pulmonary hypertension than did veterans without HIV (HR 1·34 [95% CI 1·08–1·67], p=0·0084; table 2). Baseline CD4 cell count between 200 and 499 cells per μL (HR 1·20 [1·01–1·42], p=0·035) or lower than 200 cells per μL (HR 1·32 [1·01–1·74], p=0·044) were significantly associated with incident pulmonary hypertension, but CD4 counts of 500 cells per μL or more were not (HR 1·12 (0·95–1·33), p=0·17; table 2). When modelling CD4 cell count and HIV viral load as time-varying covariates, the associations between viral control and risk of pulmonary hypertension became more pronounced. Veterans with HIV who had a CD4 count lower than 200 cells per μL or of 200–499 cells per μL had a higher risk of pulmonary hypertension than did veterans without HIV (HR 1·94 [1·49–2·54], p<0·0001, for those with <200 cell μL and HR 1·29 [1·08–1·53], p=0·0048, for those with 200–499 cells per μL; table 2). Similarly, veterans with HIV who had HIV viral loads of 500 copies per mL or more had a higher risk of pulmonary hypertension than did veterans without HIV (HR 1·88 [1·46–2·42], p<0·0001; table 2).

The associations of diabetes (interaction p=0·0082) and recreational drug use (interaction p=0·025) with incident pulmonary hypertension differed by HIV status (data not shown). Increased age, prevalent heart failure, COPD, hypertension, diabetes (in veterans with HIV and in veterans without HIV), HCV infection, current smoking, liver cirrhosis, and lower eGFR were all associated with incident pulmonary hypertension (table 3).

Results were consistent after adjustment for baseline PASP (appendix p 4). In sensitivity analyses limited to individuals without a history of heart failure or COPD at baseline (8929 veterans; 2870 with HIV and 6059 without HIV), there were 834 incident pulmonary hypertension events (290 among veterans with HIV and 544 among veterans without HIV). In this subset, 1690 individuals (534 with HIV and 1156 without HIV) developed heart failure or COPD between baseline and repeat echocardiograms. When adjusting for interim heart failure or COPD as a time-varying covariate, HIV remained significantly associated with incident pulmonary hypertension (HR 1·19 [95% CI 1·01–1·38], p=0·033; appendix p 5).

Results from the secondary analyses assessing the association between HIV and pulmonary hypertension defined as PASP higher than 40 mm Hg were nearly identical to the results from the primary analysis (appendix p 6). Examination of PASP progression yielded similar effect sizes. There were 1725 progression events (defined as an increase of at least 10 mm Hg), 561 in veterans with HIV and 1164 in veterans without HIV. PASP progression rates per 1000 person-years were higher in veterans with HIV (IR 36·4 [95% CI 33·5–39·5]) than in veterans without HIV (IR 31·9 [30·2–33·8], p=0·012). In multivariable adjusted models, HIV infection was associated with an increase in PASP progression hazard (adjusted HR 1·13 [95% CI 1·01–1·26]; p=0·026; table 4, figure 2B). In models with time-varying HIV biomarkers, low CD4 count (<200 cells per μL) and high HIV viral load (≥500 copies per mL) were associated with increased hazard of PASP progression but CD4 count of 200 cells per μL or more and an HIV viral load of less than 500 copies per mL were not (table 4).

## Discussion

This is, to our knowledge, the largest cohort study to date to examine the association between HIV infection and risk of incident echocardiographic pulmonary hypertension. We found that HIV infection had a direct effect on incident pulmonary hypertension among US veterans referred for echocardiography after adjusting for pulmonary hypertension risk factors. The risk of incident pulmonary hypertension was also present in patients with no baseline history of heart failure or COPD, the most common risk factors for pulmonary hypertension among US veterans, and persisted after accounting for interim diagnoses of heart failure or COPD between baseline and repeat echocardiograms. Finally, we observed that low CD4 count and high viral load were associated with an increased risk of pulmonary hypertension. These associations were more pronounced after modelling viral control metrics as time-varying covariates and persisted when pulmonary hypertension was defined more stringently (PASP >40 mm Hg) and when we assessed disease progression (PASP increase >10 mm Hg). These data are important for patients and practitioners because pulmonary hypertension is associated with increased mortality among individuals with HIV infection and is particularly underrecognised among veterans.^4,20^

This study builds on previous work in this area with several notable improvements in study design. First, other than our previous work in the veteran population, this study is an order of magnitude larger than previous studies on the association of HIV with pulmonary hypertension, which might have been underpowered to detect important associations. The size of our cohort allowed us to adjust for potential confounders, particularly known risk factors for pulmonary hypertension such as heart failure, COPD, diabetes, and renal dysfunction. Second, we used a longitudinal study design including data from all serial echocardiograms in the VA electronic medical record performed in our population, which allowed us to report incident pulmonary hypertension rates in HIV infected individuals for the first time, to our knowledge. IRs reported in this study probably underestimate the true burden of pulmonary hypertension in people living with HIV because we considered the absence of a repeat PASP estimate tantamount to the absence of incident pulmonary hypertension, which is conservative; furthermore, exclusion of individuals without repeat echocardiograms could introduce selection bias. Previous studies with echocardiography data have been cross-sectional; as a result, IRs and associations between viral control over time and future risk of pulmonary hypertension have not been previously reported.^21-24^ Finally, we considered HIV viral control metrics (CD4 count and viral load) as time-varying covariates, which allowed us to account for fluctuation in these factors over time rather than assuming they were constant. Previous studies have only considered viral control at the time of baseline echocardiogram or right heart catheterisation, which can introduce bias because viral control varies over time, which might have an impact on pulmonary pressure dynamics.^22,25^

Although we observed a direct association of HIV with incident pulmonary hypertension, we speculate that the most common causes of pulmonary hypertension in our cohort are due to heart failure and COPD. Heart failure was rare at the baseline echocardiogram (because we excluded individuals with PASP >35 mm Hg), but its prevalence increased significantly during follow-up. COPD is highly prevalent among veterans and was associated with incident pulmonary hypertension in our sample. Associations between pulmonary hypertension, heart failure, and COPD are not surprising, but have not previously been described in people living with HIV. It is important for practitioners to have awareness of the potential for incident pulmonary hypertension among people with HIV because rising pressure might warrant further diagnostic evaluation (eg, catheterisation and polysomnography) or more aggressive management of underlying risk factors. For example, management of diabetes might warrant particular attention in patients with HIV, given our finding of an increased risk of pulmonary hypertension among people with diabetes and HIV.

The relationship between viral control and risk of pulmonary hypertension in the literature is mixed and it has not been studied longitudinally. We previously reported that poor viral control (ie, high viral load and low CD4 count) was associated with an increased risk of prevalent pulmonary hypertension.^4^ Others have reported a lack of an association between viral control and risk of pulmonary hypertension.^22^ These studies (including our previous report)^4^ considered only measures of viral control obtained nearest in time to the assessment of pulmonary pressure, which does not capture variation in viral load or CD4 cell count over time. Our use of time-varying covariates addresses this limitation of previous work and more closely approximates the true relationship between pulmonary hypertension risk and viral control. Our results show a consistent association between high viral load, low CD4 cell count, and both the risk of reaching clinical pulmonary hypertension thresholds and PASP progression of more than 10 mm Hg. The biological basis for these observations is unknown, although several hypotheses exist as to how viral infection and associated immune dysregulation might contribute to pulmonary vascular disease. Viral proteins, particularly Nef, may contribute to pulmonary vasculopathy as evident by the development of complex vascular lesions in Rhesus monkeys infected with simian immunodeficiency virus containing the *nef* gene.^26^ Another hypothesis is activation of adaptive immunity leading to immune cell migration and inflammation in the pulmonary vasculature.^27^ Another contribution might arise from behaviours that often accompany poor medication compliance (eg, smoking and drug and alcohol misuse) and increase the risk of cardiopulmonary disease generally. We did not report antiretroviral use because treatment has changed dramatically over the course of our study and we did not expect antiretroviral effects on the pulmonary vasculature that are not mediated through HIV viraemia and CD4 cell count.

The main limitation of this study is the use of retrospective data obtained from clinically indicated echocardiograms, which may not be generalisable to asymptomatic people with HIV in the community. However, we submit that these data are highly generalisable to symptomatic individuals with HIV seeking care. We used echocardiographic estimates of pulmonary pressure because most patients with pulmonary hypertension never undergo gold-standard invasive haemodynamic assessment. Echocardiographic data in this study were extracted from clinical reports rather than interpreted in a core laboratory or performed according to a standardised protocol. We also acknowledge potential for selection bias in that patients who get referred for echocardiography might differ in important ways from those who do not. We are unable to account for the means of HIV in our sample, and we cannot rule out the possibility that our results are, in part, due to intravenous drug use among veterans with HIV. However, since only 7·4% of veterans with HIV in our sample had recorded recreational drug use and it was not associated with incident pulmonary hypertension, it is unlikely that drug use is a primary driver of pulmonary hypertension in our sample. Furthermore, cause of death is poorly captured in this sample which precludes our ability to examine death as a competing event. Finally, our cohort is predominantly male, making our results poorly generalisable to women.

HIV infection is associated with incident echocardiographic pulmonary hypertension among US veterans. This association is strongest in those with poor HIV control and appears to exist even in the absence of heart failure or COPD. These observations are important for people living with HIV and HIV providers because pulmonary hypertension is associated with increased risk of hospitalisation and mortality, might be modifiable with risk factor control, and is underdiagnosed.

## Supplementary Material

1

## Figures and Tables

**Table 1:** Baseline characteristics by HIV status

	Veterans without HIV(n=8854)	Veterans with HIV (n=4174)
Baseline PASP, mm Hg		
Mean (SD)	26·1 (6·7)	26·2 (6·6)
Median (IQR)	27·5 (22·4–31·0)	27·5 (22·5–31·3)
Number of PASPs		
Mean (SD)	2·6 (3·1)	2·7 (4·3)
Median (Q1–Q3)	2 (1–3)	2 (1–3)
Age, years		
Mean (SD)	58·2 (9·7)	57·7 (9·9)
Median (IQR)	58 (52–64)	58 (51–64)
Sex		
Male	8583 (96·9%)	4074 (97·6%)
Female	271 (3·1%)	100 (2·4%)
Race or ethnicity		
White	3531 (39·9%)	1704 (40·8%)
African American	4310 (48·7%)	1999 (47·9%)
Hispanic	773 (8·7%)	317 (7·6%)
Other	240 (2·7%)	154 (3·7%)
Prevalent heart failure	170 (1·9%)	102 (2·4%)
COPD	2705 (30·6%)	1252 (30·0%)
Hypertension*		
None	460 (5·2)	459 (11·0)
Controlled	5511 (62·3)	2632 (63·2)
Uncontrolled	2870 (32·5)	1073 (25·8)
Diabetes	3494 (39·5%)	1163 (27·9%)
Hepatitis C infection*		
Uninfected	6072 (72·0%)	2544 (63·4%)
Infected	1504 (17·8%)	1373 (34·2%)
Never tested	863 (10·2%)	97 (2·4%)
Smoking status*		
Never	2755 (32·7%)	1183 (30·3%)
Former	2115 (25·1%)	931 (23·9%)
Current	3546 (42·1%)	1790 (45·9%)
Liver cirrhosis (FIB-4>3·25)*	550 (6·4%)	534 (13·4%)
Recreational drug use	341 (3·9%)	310 (7·4%)
eGFR, mL/min per 1·73m^2^*		
Mean (SD)	85·3 (31·8)	83·4 (31·6)
Median (IQR)	84·2 (68·5–101·1)	82·6 (65·1–101·6)
BMI, kg/m^2^*		
Mean (SD)	29·9 (6·4)	26·2 (5·4)
Median (IQR)	29·2 (25·4–33·4)	25·5 (22·4–29·2)
CD4 count, cells per μL*		
Mean (SD)	..	486·6 (304·5)
Median (IQR)	..	443 (261–661)
HIV viral load, copies per mL*		
Mean (SD)	..	25 090·3 (20 8131·7)
Median (IQR)	..	50 (40–400)
Undetectable viral load†	..	1958 (54·8)
ART usage		
ART naive	..	873 (20·9%)
Previous ART	..	669 (16·0%)
Current ART	..	2632 (63·1%)
Time on ART, years‡		
Mean (SD)	..	8·5 (5·9)
Median (IQR)	..	8·2 (3·3–13·1)

All characteristics significantly differed across HIV status, via Wilcoxon tests or χ^2^ except: prevalent heart failure (p=0·0511) and COPD (p=0·5196).

ART=antiretroviral therapy. BMI=body-mass index. COPD=chronic obstructive pulmonary disease. eGFR=estimated glomerular filtration rate. FIB-4=fibrosis 4 score. PASP=pulmonary artery systolic pressure.

* All variables had complete data except hypertension (available on 8841 veterans without HIV and 4164 veterans with HIV), hepatitis C Infection (available on 8439 veterans without HIV and 4014 veterans with HIV), smoking status (available on 8416 veterans without HIV and 3904 veterans with HIV), FIB-4 (available on 8643 veterans without HIV and 3995 veterans with HIV), eGFR (available on 8784 veterans without HIV and 4148 veterans with HIV), BMI (available on 8827 veterans without HIV and 4149 veterans with HIV), CD4 cell count (available on 3558 veterans with HIV), and HIV viral load (available on 3573 veterans with HIV).

† Among those with data on baseline HIV viral load.

‡ Among those currently on ART at baseline.

**Table 2:** Rates and risk of incident pulmonary hypertension (pulmonary artery systolic pressure >35 mm Hg)

	Number of participants	Number of pulmonary hypertension events	Rate per 1000 person-years (95% CI)	Unadjusted risk of pulmonary hypertension		Adjusted risk of pulmonary hypertension*		Time-varying CD4 cell count or HIV viral loadt†	
						
				HR (95% CI)	p value	HR (95% CI)	p value	HR (95% CI)	p value
**Stratified by HIV status**									
Veterans without HIV	8854	904	23·4 (21·9–24·9)	1 (ref)	..	1 (ref)	..	..	..
Veterans with HIV	4174	468	28·6 (26·1–31·3)	1·25 (1·12–1·40)	<0·0001	1·18 (1·05–1·34)	0·0062	..	..
**Stratified by HIV status and CD4 cell count**									
Veterans without HIV	8854	904	23·4 (21·9–24·9)	1 (ref)	..	1 (ref)	..	1 (ref)	..
Veterans with HIV, CD4 ≥500 cells per μL	1513	140	25·1 (21·2–29·6)	1·18 (1·00–1·39)	0·048	1·12 (0·95–1·33)	0·17	0·95 (0·79–1·14)	0·59
Veterans with HIV, 200≤CD4<500 cells per μL	1464	167	29·0 (24·9–33·7)	1·29 (1·10–1·52)	0·0016	1·20 (1·01–1·42)	0·035	1·29 (1·08–1·53)	0·0048
Veterans with HIV, CD4 <200 cells per μL	581	62	29·8 (23·2–38·2)	1·31 (1·01–1·71)	0·0421	1·32 (1·01–1·74)	0·044	1·94 (1·49–2·54)	<0·0001
Veterans with HIV, missing CD4 cell count‡	616	99	33·9 (27·8–41·3)	..	..	..	..	..	..
**Stratified by HIV status and viral load**									
Veterans without HIV	8854	904	23·4 (21·9–24·9)	1 (ref)	..	1 (ref)	..	1 (ref)	..
Veterans with HIV, viral load <500 copies per mL	2781	276	27·0 (24·0–30·4)	1·26 (1·11–1·43)	0·0003	1·14 (0·99–1·31)	0·051	1·08 (0·94–1·24)	0·29
Veterans with HIV, viral load ≥500 copies per mL	792	92	28·5 (23·2–34·9)	1·21 (0·98–1·50)	0·082	1·34 (1·08–1·67)	0·0084	1·88 (1·46–2.42)	<0·0001
Veterans with HIV, missing viral load‡	601	100	34·7 (28·5–42·2)	..	..	..	..	..	..

COPD=chronic obstructive pulmonary disease. eGFR=estimated glomerular filtration rate. HR=hazard ratio.

* Adjusted for age sex, race or ethnicity, prevalent heart failure, COPD, hypertension, diabetes, smoking status, body-mass index, eGFR, hepatitis C virus infection, liver cirrhosis, and recreational drug use.

† Adjusted for covariates in * with time-varying HIV biomarkers.

‡ Missing category used for calculation of incidence rates. For models, missing CD4 cell counts and HIV viral loads were imputed.

**Table 3:** Predictors of incident pulmonary hypertension

	Hazard ratio (95% CI)*	p value
Age, years (per 5 years)	1·21 (1·17–1·26)	<0·0001
Male sex	0·94 (0·62–1·41)	0·69
Race or ethnicity (*vs* White)		
African American	1·07 (0·93–1·23)	0·050
Hispanic	0·66 (0·49–0·89)	0·0007
Other	0·66 (0·41–1·08)	0·13
Prevalent heart failure	1·82 (1·23–2·68)	0·0006
COPD	1·51 (1·32–1·73)	<0·0001
Hypertension (*vs* no hypertension)		
Controlled hypertension	1·74 (1·26–2·40)	0·0007
Uncontrolled hypertension	1·92 (1·38–2·67)	0·0001
Diabetest†		
Veterans without HIV	1·47 (1·28–1·68)	<0·0001
Veterans with HIV	2·02 (1·67–2·44)	<0·0001
Hepatitis C infection (*vs* no hepatitis C infection)		
Infected	1·48 (1·24–1·77)	<0·0001
Never tested	0·87 (0·69–1·09)	0·54
Smoking status (*vs* never smoker)		
Former smoker	1·12 (0·93–1·35)	0·034
Current smoker	1·26 (1·06–1·49)	0·0021
Liver cirrhosis (FIB-4 >3·25 *vs* ≤3·25)	1·56 (1·20–2·04)	<0·0001
eGFR, mL/min per 1·73m^2^ (per 10 mL/min per 1·73m^2^)	0·90 (0·88–0·93)	<0·0001
Body-mass index, kg/m^2^ (per 5 kg/m^2^)	0·99 (0·93–1·04)	0·59
Recreational drug use†		
Veterans without HIV	1·28 (0·92–1·79)	0·15
Veterans with HIV	0·72 (0·49–1·06)	0·093
CD4 cell count, cells per μL (per 200 cells per μL)‡	0·97 (0·90–1·05)	0·48
HIV viral load, copies per mL (≥500 *vs* <500)‡	1·18 (0·92–1·51)	0·20

COPD=chronic obstructive pulmonary disease. eGFR=estimated glomerular filtration rate. FIB-4=fibrosis 4 score.

* Estimates from a multivariable-adjusted model including all listed variables as covariates.

† Significant interaction between HIV status and variable, so estimates are presented by HIV status.

‡ Only in veterans with HIV.

**Table 4:** Rates and risk of PASP progression (PASP Increase >10 mm Hg)

	Number of participants	Number of progressions	Rate per 1000 person-years (95% CI)	Unadjusted risk		Adjusted risk*		Time-varying CD4 cell count or HIV viral load†	
						
				HR (95% CI)	p value	HR (95% CI)	p value	HR (95% CI)	p value
**Stratified by HIV status**									
Veterans without HIV	8854	1164	31·9 (30·2–33·8)	1 (ref)	..	1 (ref)	..	..	..
Veterans with HIV	4174	561	36·4 (33·5–39·5)	1·14 (1·03–1·26)	0·014	1·13 (1.01–1·26)	0·026	..	..
**Stratified by HIV Status and CD4 Cell Count**									
Veterans without HIV	8854	1164	31·9 (30·2–33·8)	1 (ref)	..	1 (ref)	..	1 (ref)	..
Veterans with HIV, CD4 count ≥500 cells per μL	1513	181	33·9 (29·3–39·2)	1·11 (0·95–1·28)	0·18	1·10 (0·94–1·28)	0·23	0·99 (0·84–1·17)	0·95
Veterans with HIV, 200≤CD4<500 cells per μL	1464	200	37·0 (32·3–42·6)	1·17 (1·01–1·36)	0·031	1·14 (0·98–1·34)	0·079	1·15 (0.98–1·36)	0·088
Veterans with HIV CD4 <200 cells per μL	581	68	34·7 (27·3–44·0)	1·11 (0·88–1·41)	0·39	1·19 (0·93–1·53)	0·16	1·71 (1·33–2·20)	<0·0001
Veterans with HIV, Missing CD4 cell count‡	616	112	41·1 (34·1–49·4)	..	..	..	..	..	..
**Stratified by HIV status and viral load**									
Veterans without HIV	8854	1164	31·9 (30·2–33·8)	1 (ref)	..	1 (ref)	..	1 (ref)	..
Veterans with HIV, viral load <500 copies per mL	2781	346	35·7 (32·1–39·7)	1·15 (1·03–1·29)	0·011	1·11 (0·98–1·25)	0·089	1·06(0·93–1·20)	0·39
Veterans with HIV, viral load ≥500 copies per mL	792	102	33·4 (27·5–40·5)	1·07 (0·88–1·31)	0·51	1·22 (0·99–1·50)	0·061	1·56 (1·22–1·98)	<0·0001
Veterans with HIV, missing viral load‡	601	113	42·1 (35·0–50·7)	..	..	..	..	..	..

COPD=chronic obstructive pulmonary disease. eGFR=estimated glomerular filtration rate. HR=hazard ratio. PASP=pulmonary artery systolic pressure.

* Adjusted for age sex, race or ethnicity, prevalent heart failure, COPD, hypertension, diabetes, smoking status, body-mass index, eGFR, Hepatitis C infection, liver cirrhosis, and recreational drug use.

† Adjusted for covariates in * with time-varying HIV biomarkers.

‡ Missing category used for calculation of incidence rates. For models, missing CD4 counts and HIV viral loads were imputed.

## Research in context

### Evidence before this study

We used PubMed to identify studies reporting echocardiographic estimates of pulmonary pressure among individuals with HIV with the search terms: HIV, echocardiography, and pulmonary hypertension. All published studies describing the association between echocardiographic pulmonary hypertension and HIV were cross-sectional (describing prevalence only) and most did not include appropriate comparison groups or account for viral control over time. Thus, no previous data exist incorporating longitudinal echocardiographic estimates of pulmonary pressure to derive rates of incident pulmonary hypertension among individuals with HIV.

### Added value of this study

We used data from 13 028 participants (4174 [32%] with HIV) in the Veterans Aging Cohort Study to assess the association of HIV with incident echocardiographic pulmonary hypertension. We observed that HIV is associated with higher incidence of pulmonary hypertension even after adjusting for known risk factors for pulmonary hypertension.

The association was particularly marked in those with low CD4 count and in those with high HIV viral load.

### Implications of all the available evidence

US veterans living with HIV appear to be at increased risk of pulmonary hypertension compared with demographically similar uninfected veterans. However, veterans with CD4 counts of 500 cells per μL or more or with HIV viral loads lower than 500 copies per mL did not display increased risk compared with uninfected veterans after multivariable adjustment. Thus, as with other cardiopulmonary diseases, suppression of HIV should be prioritised to lessen the burden of pulmonary hypertension in people living with HIV. These observations are important for people living with HIV and health-care providers because pulmonary hypertension is associated with increased risk of hospitalisation and mortality, might be modifiable with risk factor control, and is underdiagnosed.
