# Supplementary material for: Association between HIV and incident pulmonary hypertension in US Veterans: a retrospective cohort study
Source: Lancet Healthy Longev. Author manuscript; Available in PMC 2021 Jul 21. (PMC8294078; doi:10.1016/s2666-7568(21)00116-1)
Supplement: 1 [file NIHMS1720506-supplement-1.pdf]

THE LANCET
Healthy Longevity

Supplementary appendix

This appendix formed part of the original submission and has been peer reviewed. We post it as supplied by the authors.

Supplement to: Duncan MS, Alcorn CW, Freiberg MS, et al. Association between HIV and incident pulmonary hypertension in US Veterans: a retrospective cohort study. *Lancet Healthy Longev* 2021; published online June 16. https://doi.org/10.1016/S2666-7568(21)00116-1.

Supplemental Material

Additional Details on Covariate Definitions

Age, sex, and race/ethnicity data were obtained from administrative data. History of heart failure, chronic obstructive pulmonary disease (COPD), and recreational drug use were determined using ICD-9 codes as outlined in previous VACS publications.(1–3) These comorbidities were considered prevalent if present at any time prior to and up to 180 days after the baseline echocardiogram. Hypertension was categorized as absent (blood pressure < 140/90 mm Hg and no antihypertensive medication), controlled (blood pressure < 140/90, on antihypertensive medication), or uncontrolled (blood pressure ≥140/90 mm Hg regardless of antihypertensive medication use).(4) Body mass index (BMI) and smoking status were extracted from the VA health factors data repository.(5) Diabetes mellitus is based on ICD-9 codes as well as laboratory measured fasting glucose as previously described.(6) Chronic kidney disease and liver cirrhosis were captured via estimated glomerular filtration rate (eGFR) and fibrosis-4 score (FIB-4), respectively, from outpatient lab data.(6) We extracted BMI, smoking status, eGFR, and FIB-4 values closest to date of baseline echocardiogram in a window of beginning of VA electronic medical record to baseline + 180 days. Liver cirrhosis was defined as FIB-4>3.25. Hepatitis C virus (HCV) infection was categorized as “positive”, “negative”, or “never tested” based on detectable HCV RNA, positive antibody test, and/or documented diagnosis.(7,8)

E-REFERENCES

1. Fultz SL, Skanderson M, Mole LA, Gandhi N, Bryant K, Crystal S, et al. Development and verification of a "virtual" cohort using the national VA health information system [Internet]. Vol. 44, Medical Care. Med Care; 2006 [cited 2021 Apr 26]. Available from: https://pubmed.ncbi.nlm.nih.gov/16849965/

2. Crothers K, Huang L, Goulet JL, Goetz MB, Brown ST, Rodriguez-Barradas MC, et al. HIV infection and risk for incident pulmonary diseases in the combination antiretroviral therapy era. Am J Respir Crit Care Med [Internet]. 2011 Feb 1 [cited 2021 Apr 27];183(3):388–95. Available from: /pmc/articles/PMC3266024/

3. Maron BA, Hess E, Maddox TM, Opotowsky AR, Tedford RJ, Lahm T, et al. Association of borderline pulmonary hypertension with mortality and hospitalization in a large patient cohort: Insights from the Veterans Affairs clinical assessment, reporting, and tracking program. Circulation [Internet]. 2016 Mar 29 [cited 2021 Apr 26];133(13):1240–8. Available from: https://pubmed.ncbi.nlm.nih.gov/26873944/

4. Chobanian A V., Bakris GL, Black HR, Cushman WC, Green LA, Izzo JL, et al. Seventh report of the Joint National Committee on Prevention, Detection, Evaluation, and Treatment of High Blood Pressure [Internet]. Vol. 42, Hypertension. Hypertension; 2003 [cited 2021 Apr 26]. p. 1206–52. Available from: https://pubmed.ncbi.nlm.nih.gov/14656957/

5. McGinnis KA, Brandt CA, Skanderson M, Justice AC, Shahrir S, Butt AA, et al. Validating smoking data from the Veteran's affairs health factors dataset, an electronic data source. Nicotine Tob Res [Internet]. 2011 Dec 12 [cited 2021 Apr 26];13(12):1233–9. Available from: www.vacohort.org.

6. Butt AA, McGinnis K, Rodriguez-Barradas MC, Crystal S, Simberkoff M, Goetz MB, et al. HIV infection and the risk of diabetes mellitus. AIDS [Internet]. 2009 Jun 19 [cited 2021 Apr 26];23(10):1227–34. Available from: /pmc/articles/PMC2752953/

7. Tate JP, Justice AC, Hughes MD, Bonnet F, Reiss P, Mocroft A, et al. An internationally generalizable risk index for mortality after one year of antiretroviral therapy. AIDS [Internet]. 2013 Feb 20 [cited 2021 Apr 26];27(4):563–72. Available from: https://europepmc.org/articles/PMC4283204

8. Tate JP, Sterne JAC, Justice AC. Albumin, white blood cell count, and body mass index improve discrimination of mortality in HIV-positive individuals. AIDS [Internet]. 2019 Apr 1 [cited 2021 Apr 26];33(5):903–12. Available from: https://pubmed.ncbi.nlm.nih.gov/30649058/

Table E1. Rates and Risk of Incident Pulmonary Hypertension (PASP > 35mmHg) additionally adjusted for Baseline PASP

Group	N	PH Events	Rate/1000PY [95% CI]	Minimally Adjusted PH Risk*		Adjusted PH Risk†			
Stratified by HIV Status									
				HR [95% CI]	p value	HR [95% CI]	p value		
Veterans without HIV	8854	904	23·4 [21·9, 24·9]	1·00	··	1·00	··		
Veterans with HIV	4174	468	28·6 [26·1, 31·3]	1·25 [1·12, 1·40]	<0·0001	1·17 [1·04, 1·32]	0·012		
Stratified by HIV Status and CD4 Cell Count									
**Group**	**N**	**PH Events**	**Rate/1000PY [95% CI]**	**Minimally Adjusted PH Risk***		**Adjusted PH Risk†**		**Time-Varying CD4 Cell Count‡**	
				**HR [95% CI]**	**p value**	**HR [95% CI]**	**p value**	**HR [95% CI]**	**p value**
Veterans without HIV	8854	904	23·4 [21·9, 24·9]	1·00	··	1·00	··	1·00	··
VWH, CD4≥500	1513	140	25·1 [21·2, 29·6]	1·19 [1·01, 1·40]	0·042	1·12 [0·95, 1·33]	0·19	0·94 [0·78, 1·13]	0·53
VWH, 200≤CD4<500	1464	167	29·0 [24·9, 33·7]	1·29 [1·10, 1·51]	0·0019	1·18 [0·99, 1·40]	0·055	1·26 [1.09, 1·50]	0·0095
VWH, CD4<200	581	62	29·8 [23·2, 38·2]	1·30 [1·00, 1·69]	0·049	1·28 [0·97, 1·68]	0·078	1·89 [1·44, 2·47]	<0·0001
VWH, Missing CD4§	616	99	33·9 [27·8, 41·3]	··	··	··	··	··	··
Stratified by HIV Status and Viral Load									
**Group**	**N**	**PH Events**	**Rate/1000PY [95% CI]**	**Minimally Adjusted PH Risk***		**Adjusted PH Risk†**		**Time-Varying HIV Viral Load‡**	
				**HR [95% CI]**	**p value**	**HR [95% CI]**	**p value**	**HR [95% CI]**	**p value**
Veterans without HIV	8854	904	23·4 [21·9, 24·9]	1·00	··	1·00	··	1·00	··
VWH, VL<500	2781	276	27·0 [24·0, 30·4]	1·27 [1·12, 1·44]	0·0002	1·14 [0·99, 1·30]	0·063	1·06 [0·92, 1·22]	0·41
VWH, VL ≥500	792	92	28·5 [23·2, 34·9]	1·18 [0·96, 1·47]	0·12	1·29 [1·04, 1·61]	0·024	1·85 [1·44, 2.38]	<0·0001
VWH, Missing VL§	601	100	34·7 [28·5, 42·2]	··	··	··	··	··	··

CI = confidence interval; HIV = human immunodeficiency virus; HR = hazard ratio; PH = pulmonary hypertension; PY = person years; VWH = Veterans with HIV

*adjusted for baseline PASP

† adjusted for baseline PASP, age sex, race/ethnicity, prevalent heart failure, COPD, hypertension, diabetes mellitus, smoking status, BMI, eGFR, HCV, liver cirrhosis, and recreational drug use.

‡ adjusted for covariates in † with time-varying HIV biomarkers

§ missing category used for calculation of incidence rates. For models, missing CD4 cell counts and HIV viral loads were imputed.

Table E2. Rates and Risk of Incident Pulmonary Hypertension (PASP > 35mmHg) in people free of HF and COPD

Stratified by HIV Status									
**Group**	**N**	**PH Events**	**Rate/1000PY [95% CI]**	**Interim HF/COPD Adjusted PH Risk**		**Multivariable Adjusted PH Risk***			
				**HR [95% CI]**	**p value**	**HR [95% CI]**	**p value**		
Veterans without HIV	6059	544	19·4 [17·8, 21·1]	1·00	---	1·00	---		
Veterans with HIV	2870	290	24·6 [21·9, 27·6]	1·29 [1·12, 1·49]	0·0005	1·19 [1·01, 1·38]	0·033		
Stratified by HIV Status and CD4 Cell Count									
**Group**	**N**	**PH Events**	**Rate/1000PY [95% CI]**	**Interim HF/COPD Adjusted PH Risk**		**Multivariable Adjusted PH Risk***		**Time-Varying CD4 Cell Count†**	
				**HR [95% CI]**	**p value**	**HR [95% CI]**	**p value**	**HR [95% CI]**	**p value**
Veterans without HIV	6059	544	19·4 [17·8, 21·1]	1·00	---	1·00	---	1·00	---
VWH, CD4≥500	1034	89	22·5 [18·3, 27·7]	1·29 [1·04, 1·59]	0·019	1·15 [0·97, 1·36]	0·11	1·06 [0·86, 1·31]	0·59
VWH, 200≤CD4<500	1008	101	23·9 [19·7, 29·1]	1·23 [0·99, 1·51]	0·054	1·17 [0·99, 1·38]	0·071	1·18 [0·96, 1·46]	0·12
VWH+, CD4<200	410	38	24·8 [18·0, 34·0]	1·33 [0·95, 1·85]	0·097	1·32 [0·99, 1·75]	0·053	1·87 [1·37, 2·56]	<0·0001
VWH, Missing CD4‡	418	62	29·6 [23·1, 38·0]	---	---	---	---	---	---
Stratified by HIV Status and Viral Load									
**Group**	**N**	**PH Events**	**Rate/1000PY [95% CI]**	**Interim HF/COPD Adjusted PH Risk**		**Multivariable Adjusted PH Risk***		**Time-Varying HIV Viral Load†**	
				**HR [95% CI]**	**p value**	**HR [95% CI]**	**p value**	**HR [95% CI]**	**p value**
Veterans without HIV	6059	544	19·4 [17·8, 21·1]	1·00	---	1·00	---	1·00	---
VWH, VL<500	1901	167	22·5 [19·4, 26·2]	1·26 [1·07, 1·47]	0·0050	1·13 [0·95, 1·34]	0·15	1·12 [0·95, 1·33]	0·19
VWH, VL ≥500	559	60	25·9 [20·1, 33·4]	1·30 [1·01, 1·68]	0·045	1·38 [1·06, 1·81]	0·019	1·35 [1·04, 1·75]	0·024
VWH, Missing VL‡	410	63	30·3 [23·7, 38·8]	---	---	---	---	---	---

CI = confidence interval; HIV = human immunodeficiency virus; HR = hazard ratio; PH = pulmonary hypertension; PY = person years

* adjusted for interim HF/COPD, age, sex, race/ethnicity, diabetes mellitus, smoking status, body mass index, eGFR, HCV, and liver cirrhosis.

† adjusted for covariates in * with time-varying HIV biomarkers

‡ missing category used for calculation of incidence rates. For models, missing CD4 cell counts and HIV viral loads were imputed

Table E3. Rates and Risk of Incident Pulmonary Hypertension (PASP > 40mmHg)

Stratified by HIV Status										
Group	N	PH Events	Rate/1000PY [95% CI]	Unadjusted PH Risk		Adjusted PH Risk*				
				HR [95% CI]	p value	HR [95% CI]	p value			
Veterans without HIV	8854	630	16.3 [15.1, 17.6]	1.00	---	1.00	---			
Veterans with HIV	4174	335	20.5 [18.4, 22.8]	1.29 [1.13, 1.47]	0·0002	1.23 [1.06, 1.42]	0·0050			
Stratified by HIV Status and CD4 Cell Count										
Group	N	PH Events	Rate/1000PY [95% CI]	Unadjusted PH Risk		Adjusted PH Risk*		Time-Varying CD4 Cell Count†		
				HR [95% CI]	p value	HR [95% CI]	p value	HR [95% CI]	p value	
Veterans without HIV	8854	630	16.3 [15.1, 17.6]	1.00	---	1.00	---	1.00	---	
VWH, CD4≥500	1513	97	17.4 [14.2, 21.2]	1.21 [0.99, 1.47]	0·064	1.17 [0.95, 1.44]	0·13	0.97 [0.78, 1.21]	0·80	
VWH, 200≤CD4<500	1464	121	21.0 [17.6, 25.1]	1.35 [1.12, 1.62]	0·0020	1.25 [1.02, 1.53]	0·029	1.39 [1.14, 1.70]	0·001	
VWH+, CD4<200	581	44	21.1 [15.7, 28.4]	1.33 [0.98, 1.81]	0·066	1.35 [0.98, 1.85]	0·067	2.06 [1.51, 2.82]	<0·0001	
VWH, Missing CD4‡	616	73	25.0 [19.9, 31.4]	---	---	---	---	---	---	
Stratified by HIV Status and Viral Load										
Group	N	PH Events	Rate/1000PY [95% CI]	Unadjusted PH Risk		Adjusted PH Risk*		Time-Varying HIV Viral Load†		
				HR [95% CI]	p value	HR [95% CI]	p value	HR [95% CI]	p value	
Veterans without HIV	8854	630	16.3 [15.1, 17.6]	1.00	---	1.00	---	1.00	---	
VWH, VL<500	2781	197	19.3 [16.7, 22.1]	1.31 [1.13, 1.51]	0·0004	1.20 [1.02, 1.40]	0·027	1.16 [0.98, 1.37]	0·080	
VWH, VL ≥500	792	65	20.1 [15.8, 25.6]	1.22 [0.95, 1.57]	0·12	1.35 [1.04, 1.76]	0·022	1.83 [1.35, 2.49]	<0·0001	
VWH, Missing VL‡	601	73	25.3 [20.1, 31.8]	---	---	---	---	---	---	

CI = confidence interval; HIV = human immunodeficiency virus; HR = hazard ratio; PH = pulmonary hypertension; PY = person years

* adjusted for age sex, race/ethnicity, prevalent heart failure, COPD, hypertension, diabetes mellitus, smoking status, body mass index, eGFR, HCV, liver cirrhosis, and recreational drug use.

† adjusted for covariates in * with time-varying HIV biomarkers

‡ missing category used for calculation of incidence rates. For models, missing CD4 cell counts and HIV viral loads were imputed
